# The Structural Design, Kinematics, and Workspace Analysis of a Novel Rod–Cable Hybrid Cable-Driven Parallel Robot

**DOI:** 10.3390/biomimetics10010004

**Published:** 2024-12-25

**Authors:** Jinrun Li, Yangmin Li

**Affiliations:** Department of Industrial and Systems Engineering, The Hong Kong Polytechnic University, Kowloon, Hong Kong SAR, China; jinrun.li@connect.polyu.hk

**Keywords:** cable-driven parallel robot, kinematic, workspace

## Abstract

This study presents a novel rod–cable hybrid planar cable-driven parallel robot inspired by the biological synergy of bones and muscles. The design integrates rigid rods and flexible cables to enhance structural stability and precision in motion control. The rods emulate bones, providing foundational support, while the cables mimic muscles, driving motion through coordinated tension. This design enables planar motions with three degrees of freedom, and a structural configuration that mitigates sagging and vibration for improved stability and accuracy by introducing rigid structure. The study develops detailed kinematic models, including Jacobian analysis for motion control, and evaluates the workspace using geometric and Monte Carlo methods.

## 1. Introduction

Cable-driven parallel robots (CDPRs) have become indispensable in modern industry, primarily due to their extensive workspace, impressive payload-to-weight ratio, and cost-efficient manufacturing processes [1,2,3,4]. Unlike traditional rigid-link robots, CDPRs use flexible cables for movement and support, enabling these systems to cover large-scale areas with greater ease. As industries like logistics, construction, and aerospace rapidly develop, the demand for robots that offer high-speed, precise, and large-scale manipulation has grown, positioning CDPRs as an ideal solution [5,6]. Their ability to transport heavy loads with precision and operate over expansive areas makes them particularly suitable for applications such as automated manufacturing lines, infrastructure inspection and maintenance, and handling of materials on a large scale [7,8].

However, the inherent flexibility and elasticity of the cables introduce unique challenges. Unlike rigid-link mechanisms, CDPRs are susceptible to complex dynamics, including cable sagging, oscillations, and non-linearities [9,10,11,12]. These factors create significant complexities in robot design, requiring sophisticated control strategies and advanced path planning algorithms to achieve reliable, accurate, and safe operations. Additionally, the need for precise cable tension management and vibration suppression demands innovative approaches, particularly as these robots are expected to operate in diverse and sometimes challenging environments.

The CDPRs are categorized based on their tensioning methods into suspended and fully constrained types. Suspended CDPRs are typically designed with fewer cables than degrees of freedom (DOFs) in the end-effector. This configuration enables the end-effector to be supported against gravity by the cables while retaining some uncontrolled DOFs. As there are no cables positioned beneath the mobile platform, suspended CDPRs are commonly utilized to achieve larger workspace areas. However, due to gravitational constraints, their operation is generally limited to static conditions [13]. Consequently, suspended CDPRs are suited to applications that require expansive workspaces but do not prioritize high precision, such as camera positioning, large-scale logistics, and transportation tasks. Conversely, in fully constrained CDPRs, the number of cables is equal to or exceeds the DOFs of the end-effector, allowing for precise control over each movement axis. This design ensures that each DOFs is actively constrained by the cables, keeping them under constant tension, thereby enhancing system stability and mitigating issues like unwanted swinging or loss of control. Fully constrained CDPRs are thus ideal for high-speed and high-precision applications, including precision manufacturing, medical rehabilitation systems, and construction assembly processes [14].

The presence of flexible cables introduces significant complexity into the modeling and analysis of CDPRs. Given the unilateral properties of cables, they must consistently remain under tension to function effectively [15]. For CDPRs with idealized cables assumed to be massless and inelastic, solving the inverse kinematics (IKs) is generally simpler than the forward kinematics (FKs), especially in parallel configurations. However, when considering real cables with notable shape and flexibility, the IKs problem becomes much more complex. In these cases, both the position parameters and cable tensions are unknown, and certain equations involved are non-algebraic, further complicating the analysis.

The workspace of CDPRs is also a critical area of research. It is heavily influenced by the tension in the cables, which directly impacts the robot’s overall performance [16]. Extensive research has been dedicated to optimizing the workspace to enhance CDPRs’ operational efficiency and application versatility. For example, a generalized ray-based lattice generation and graph representation method has been developed for arbitrary CDPRs, allowing for optimized visualization and analysis of the wrench-closure workspace (WCW) through graph theory applications [17]. Zhang [18] introduced a non-redundant translational 3-DOFs CDPR, incorporating passive springs with driving cables to support high-speed motion. By applying force-closure and geometric constraint analyses, this approach defines a cylindrical operational workspace where all cables maintain positive tension, optimized through design parameters. For tasks requiring expansive coverage, such as large-area painting, innovative workspace optimization and base-movement strategies have been proposed, with the introduction of the Inverse Reachability Workspace Ratio (IRWR) index providing a framework for selecting ideal robot configurations based on operational needs [19]. Additionally, methods have been explored for calculating horizontal cross-sections of the workspace under the assumption of a fixed platform orientation [20]. These include computationally intensive algorithms tailored for both non-deformable cables and cables with negligible mass elasticity. While accurate, these methods still require further simplification for practical application. Further advancements include a geometric method for evaluating the workspace under minimum tension conditions, enhancing computational efficiency, and optimizing tension distribution through convex analysis [21]. A differential workspace hull based on workspace triangulation has also been proposed to rapidly and accurately determine critical attributes such as volume and surface area [22]. In addition, Wang [23] explored feasible dynamic workspaces under varying acceleration, an approach valuable for high-speed precision applications in automated production and assembly.

With the increasing demand for precision treatment and efficient rehabilitation in modern medicine, the need for intelligent and flexible medical devices has become a major research focus. Traditional rigid mechanisms often face limitations when applied to areas like surgical robotics, rehabilitation devices, and assistive treatment tools. CDPRs stand out as a promising solution due to their unique features such as high precision, multi-DOFs motion, large workspace, and lightweight structure. By utilizing flexible cables for actuation, CDPRs offer unmatched flexibility and cost-effectiveness across various application scenarios [24,25]. In [26], the authors present a flexible CDPR designed for minimally invasive surgery (MIS), featuring innovative folded pouch actuators and a pneumatic inflatable scaffold for compactness, precision, and flexibility. The robot provides five degrees of freedom and is particularly suited for natural orifice procedures like colorectal surgeries. Its Bowden cable-free design eliminates friction and hysteresis issues common in traditional systems, while the inflatable scaffold enhances structural rigidity, addressing deformation and flexibility challenges in soft robotic systems. Experimental validation demonstrates exceptional performance in precision, stability, and adaptability, offering an innovative solution for robotic applications in MIS and rehabilitation devices. In [27], the authors investigate a cable-driven parallel robot for lower limb rehabilitation (CDLR) and propose a method to evaluate its stability. By considering factors such as traction point position, cable tension, system stiffness, and velocity influence function, the static and dynamic stability indices are defined. In [28], the authors propose a multi-objective optimization design method for a CDPR intended for upper limb rehabilitation tasks. Using a genetic algorithm, the topology and dimensional parameters were optimized concurrently, considering objectives such as minimizing cable tension, reducing occupied volume, and ensuring patient safety. Four LARM Wire-driven Exercising device (LAWEX) robot designs were evaluated, with the 2–2 topology identified as the optimal solution. Compared to the existing prototype, this design reduced the occupied volume by approximately 85% and lowered the maximum cable tension by 2%, achieving a balanced tradeoff between performance and safety. Currently, the CDPR mainly adopts the design of pure cable connection and uses the flexible cable as the only driving and supporting structure. This approach offers advantages such as lightweight construction, large workspaces, and low manufacturing costs, making it suitable for large-scale operational scenarios. However, the pure cable design lacks rigid support, leading to significant challenges in dynamic modeling and control. Therefore, this study introduces a novel planar CDPR, which significantly enhances the rigidity of the CDPR and effectively addresses these challenges.

The remaining parts of this paper are arranged as follows. The architecture design of this novel rod–cable hybrid CDPR will be introduced in Section 2. Section 3 will provide the kinematics analysis of the proposed planar CDPR. In addition, the workspace analysis of the proposed planar CDPR will be conducted in Section 4.

## 2. Robot Structural Design

**Figure 1:**
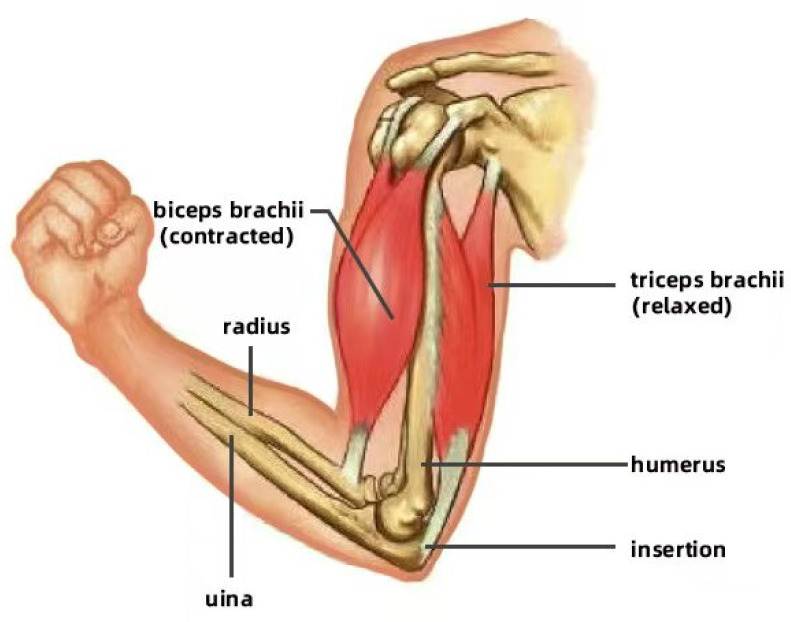
Contraction of forearm.

**Figure 2:**
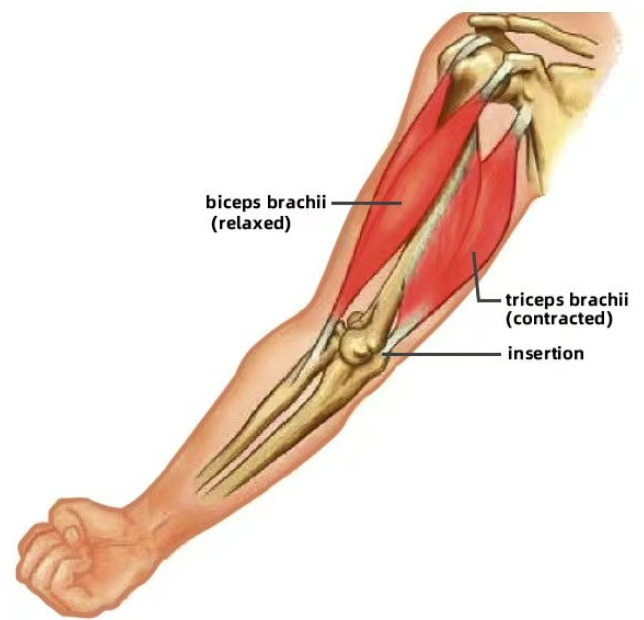
Relaxation of forearm.

In biological systems, bones provide structural support and stability for the entire body, enabling muscles to exert their maximum efficiency during contraction. For instance, in the arm, the humerus, radius, and ulna form a solid framework that allows muscles to attach to the bones and facilitate flexible movement. In addition, muscles drive the motion of bones through contraction and relaxation, with different muscle groups coordinating to achieve complex movements. For example, the biceps and triceps work antagonistically to control the bending and extending of the elbow by contracting and relaxing in opposition. Figure 1 illustrates the forearm flexion; the biceps brachii contracts to pull the forearm upward, while the triceps brachii remains relaxed. Conversely, Figure 2 displays forearm extension. The triceps brachii contracts to straighten the forearm, while the biceps brachii relaxes.

Moreover, the bones and muscles do not function independently but operate collaboratively. Bones offer stable anchor points, while muscles apply tensile forces to drive movement. This collaboration ensures efficient and stable motion. Furthermore, the bone–muscle system enables joints to possess multi-DOFs motion capabilities. For example, the shoulder and wrist joints can move flexibly in various directions due to the support provided by bones and the coordination of multiple muscle groups. This seamless collaboration between bones and muscles supports the body’s ability to perform highly dynamic and adaptable movements, integrating stability, precision, and flexibility into a unified system.

This new type of CDPR is inspired by the biological principles of bones and muscles, particularly in terms of support and drive mechanisms. It can provide efficient and flexible motion control by using this structure design.

The design of the novel CDPR incorporates rigid rods to simulate the supporting function of bones, providing a foundational structural framework for the system. These rods not only offer stability but also connect various driving points, enabling the efficient transmission of cable tension to the platform for precise positioning and stable movement. Meanwhile, the cables act as “muscles” in the system, controlling platform motion by adjusting their length. The coordinated control of cables in multiple directions allows the platform to achieve precise motion within multiple DOFs. The regulation of cable tension mimics the contraction force of muscles, enabling control over the direction and magnitude of movement. The combination of rods and cables emulates the synergistic mechanism of bones and muscles, with rigid rods providing bone-like support and cables driving the motion of the rods and platform through tensile forces. This integration ensures efficient tension transmission, resulting in more stable and precise driving performance.

**Figure 3:**
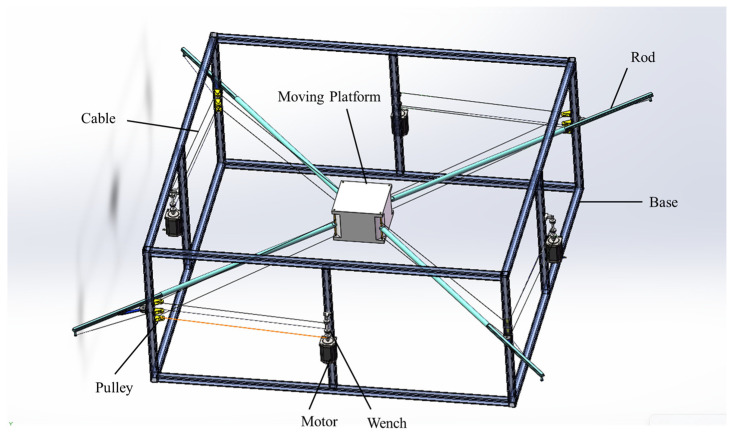
The proposed novel planar CDPR CAD model.

**Figure 4:**
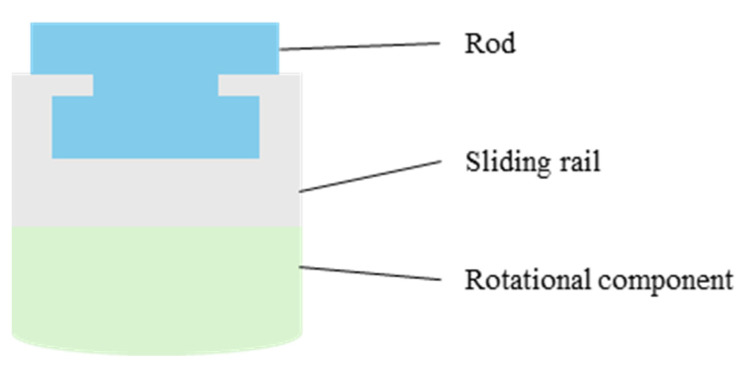
Cross-section of the rotatable sliding rail mechanism.

Figure 3 illustrates the CAD model of the proposed novel CDPR structure. Each vertical edge of the moving platform is connected by two cables and a rod, with the other end of the rod connected by an additional cable. This configuration effectively makes each vertical edge of the moving platform driven by three cables and one rod. The three cables are collectively attached to the motor’s rotating shaft, with the two cables directly connected to the moving platform sharing the same winding direction, while the cable connected to the rod is wound in the opposite direction. This design allows the rod to be pulled in coordination with the two cables connected to the moving platform. All three cables pass through three pulleys fixed on the base to change direction, with the cable connected to the rod oriented oppositely to the other two cables and attached to the rod’s end. In this structure, the three cables remain parallel and, after passing through the pulleys, they stay aligned with the rod. One end of the rod connects to the moving platform, while the other end connects to a cable. The base also provides a rotatable sliding rail mechanism, allowing the rod to rotate about the z-axis and move freely in the x–y plane. This setup forms a novel planar cable-driven parallel robot that combines cables and rods, achieving three degrees of freedom: translational motion in the x–y plane and rotational motion about the z-axis. Figure 4 provides the cross-section of the rotatable sliding rail mechanism; the entire mechanism is designed in a form resembling a cylinder. The green component represents a rotatable structure designed to provide rotational motion in the x–y plane. The gray component is a sliding rail mechanism mounted on top of the rotatable structure. It is securely attached to the green structure and rotates together with it, maintaining alignment throughout the rotational motion. The blue component is a rod that can freely slide along the gray sliding rail, allowing translational movement within the constraints of the rail.

This novel CDPR design effectively addresses the common issues of sagging and vibration in traditional systems while significantly enhancing overall rigidity. By incorporating rigid rods, the system gains additional structural support, reducing deformation caused by gravity or external loads and ensuring precise positioning even under dynamic conditions. The rods can be used to distribute cable tensions, mitigating platform sagging and improving stability during high-speed or heavy-load operations. Furthermore, the rigid rods act as dampers, suppressing oscillations and minimizing vibration caused by tension fluctuations during motion, resulting in smoother transitions and improved control accuracy. This integration of rigid rods and cable control significantly improves the CDPR’s performance, making it more robust, stable, and reliable for complex tasks.

## 3. Kinematics Analysis of the Planar CDPR

### 3.1. Kinematics of the Planar CDPR

**Figure 5:**
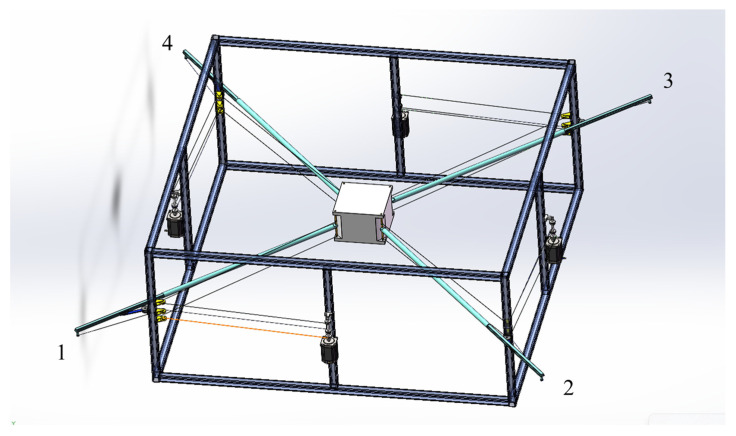
The four groups of the proposed planar CDPR.

**Figure 6:**
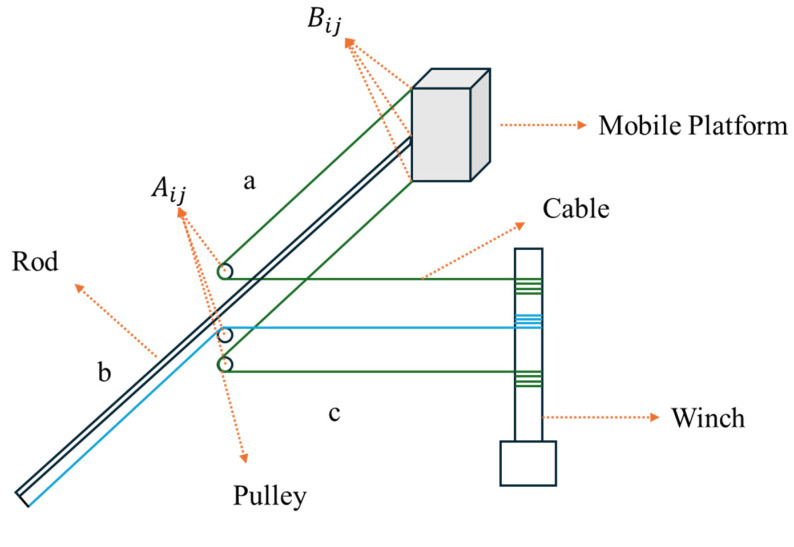
The i-th group of the proposed planar CDPR.

The proposed planar CDPR structure consists of four connection groups, denoted as $i\in \left\{1,2,3,4\right\}$. Figure 5 illustrates how the proposed planar CDPR has been divided by four groups. Each group is composed of three layers—upper, middle, and lower, denoted by the letter j, where j belongs to {a, b, c}. Figure 6 displays the i-th group structure of the proposed planar CDPR. The pulley and rotatable sliding rail mechanism are fixed on the base of the platform. The middle cable b is connected to the rod and serves the function of driving and supporting the rod, while the upper and lower cables a and c are directly connected to the moving platform, providing direct drive and control to the platform. To precisely describe the positional relationships within the structure, we define the center of each pulley as $A_{ij}$, where i indicates the i-th connection group, and j indicates the layer within that group. In addition, the points where the cables and rod contact the moving platform are defined as $B_{ij}$, identifying specific connection locations on the platform. Therefore, the coordinates of $A_{ij}$ and $B_{ij}$ can be represented as ($a_{ijx}$, $a_{ijy}$) and ($b_{ijx}$, $b_{ijy}$), respectively. Moreover, each pulley has the same size, with its radius denoted as $r_{p}$.

**Figure 7:**
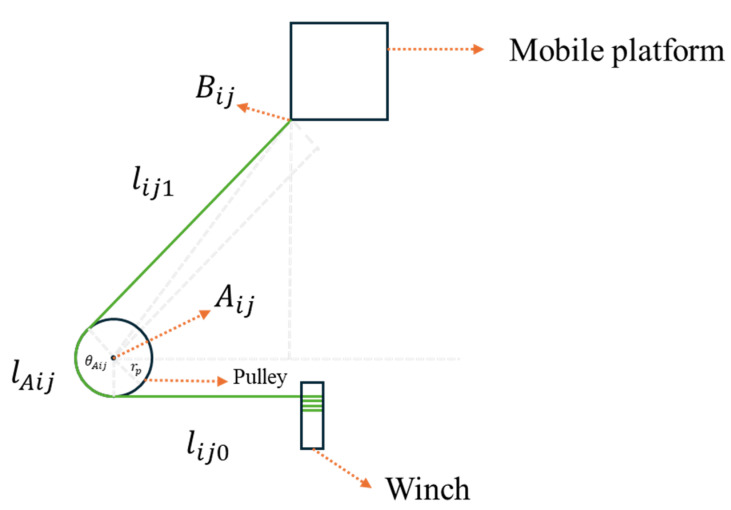
The lower and upper cables’ connection in the proposed novel planar CDPR.

The lower and upper cables in this system are directly connected to the vertical edges of the moving platform, providing the primary driving force and positional control for the platform. Figure 7 illustrates the lower and upper cables’ connection in the proposed novel planar CDPR. To control the CDPR, the cable length of each cable needs to be determined based on the position of the moving platform. For the cable of the lower and upper layers, the cable length can be represented as follows:

(1) $$l_{ij}=l_{Aij}+l_{ij0}+l_{ij1},$$

where $l_{Aij}$ is the contact length between the pulley and the cable, and $l_{ij0}$ is the cable length between the winch and the pulley, which is a constant value. In addition, $l_{ij1}$ is the distance from the pulley to the connection point between the moving platform and the cable. Since the coordinates of the pulley center $A_{ij}$, the pulley radius $r_{p}$, and the coordinates of the connection point $B_{ij}$ between the moving platform and the cable are known, we can directly obtain the length of $l_{ij1}$:

(2) $$l_{ij1}=\sqrt{{\left(a_{ijx}-b_{ijx}\right)}^{2}+{\left(a_{ijy}-b_{ijy}\right)}^{2}-r_{p}^{2}}.$$

In terms of the cable contact angle between the pulley and the cable, the angle $\theta_{Aij}$ can be represented as follows:

(3) $$\theta_{Aij}=\pi -\left(\arctan\left(\frac{b_{ijy}-a_{ijy}}{b_{ijx}-a_{ijx}}\right)-\arctan\left(\frac{r_{p}}{l_{ij1}}\right)\right).$$

Therefore, the cable contact length between the pulley and the cable $l_{Aij}$ can be described as follows:

(4) $$l_{Aij}=r_{p}\theta_{Aij}.$$

After combining Equations (1)–(4), the expression of upper and lower cable length can be obtained:

(5) $$l_{ij}=l_{ij0}+\left(\pi -\arctan\left(\frac{b_{ijy}-a_{ijy}}{b_{ijx}-a_{ijx}}\right)+\arctan\left(\frac{r_{p}}{l_{ij1}}\right)\right)r_{p}+\sqrt{{\left(a_{ijx}-b_{ijx}\right)}^{2}+{\left(a_{ijy}-b_{ijy}\right)}^{2}-r_{p}^{2}}.$$

**Figure 8:**
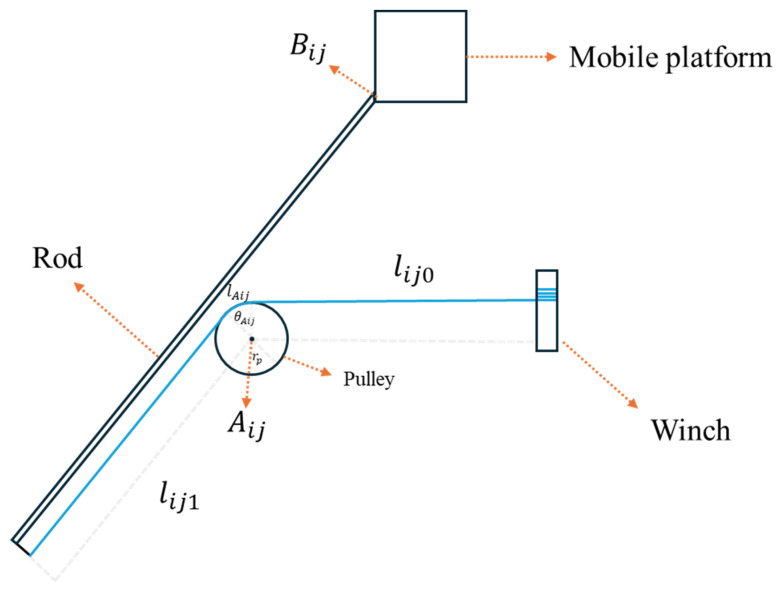
The middle cable’s connection in the proposed novel planar CDPR.

In this system, the design of the rod connections is crucial for motion control and stability. Figure 8 displays the middle cable’s connection in the proposed novel planar CDPR. Each rod is fixed at both the rotatable sliding rail on the base and the moving platform, driven by the middle cable, and it remains parallel to the upper and lower cables directly connected to the moving platform. This arrangement not only provides structural support but also ensures smooth and stable planar movement of the platform. Because each rod remains parallel to the platform-connected cables, this configuration simplifies the calculation of the middle cable length, allowing for efficient control of the moving platform position. Furthermore, this parallel alignment enhances the overall rigidity and precision of the system, reducing undesired motion fluctuations and improving positional accuracy. Assuming each rod has a fixed length of $L_{rod}$, we can express the length of the middle cable as follows:

(6) $$l_{ib}=l_{Aib}+l_{ib0}+l_{ib2},$$

where $l_{ib2}$ refers to the length of the cable from the pulley to the point where it connects with the end of the rod. The value of $l_{ib0}$ should be the same as that of $l_{ij0}$, since the three cables in each group remain parallel, and the position of the pulley is fixed. Therefore, $l_{ib2}$ can be represented as follows:

(7) $$l_{ib2}=L_{rod}-l_{ij1},$$

where j belongs to {a, c} in this equation. In addition, the cable contact angle between the pulley and the cable $\theta_{Aib}$ can be represented as follows:

(8) $$\theta_{Aib}=\pi -\theta_{Aij},$$

where j belongs to {a, c} in this equation. Since the cable and the other two cables wrap around opposite sides of the pulley and remain parallel yet oppositely directed after passing over it, their contact arc angles with the pulley are complementary, summing to $180^{{}^{\circ}}$. This complementary angular relationship arises from the fixed positioning of the pulley, which enforces the geometrical condition necessary for the cables to remain parallel yet oppositely directed, preserving the system’s spatial symmetry and alignment. Therefore, the middle cable contact length between the pulley and the cable $l_{Aib}$ should be described as follows:

(9) $$l_{Aib}=r_{p}\theta_{Aib}.$$

By integrating Equations (2), (3), and (6)–(9), we can derive the expression of the middle cable length:

(10) $$l_{ib}=\left(\arctan\left(\frac{b_{ijy}-a_{ijy}}{b_{ijx}-a_{ijx}}\right)+\arctan\left(\frac{r_{p}}{l_{ij1}}\right)\right)r_{p}+l_{ib0}+L_{rod}-\sqrt{{\left(a_{ijx}-b_{ijx}\right)}^{2}+{\left(a_{ijy}-b_{ijy}\right)}^{2}-r_{p}^{2}},$$

where j is equal to b in this equation.

### 3.2. Jacobian Matrix of the Proposed Novel Planar CDPR

**Figure 9:**
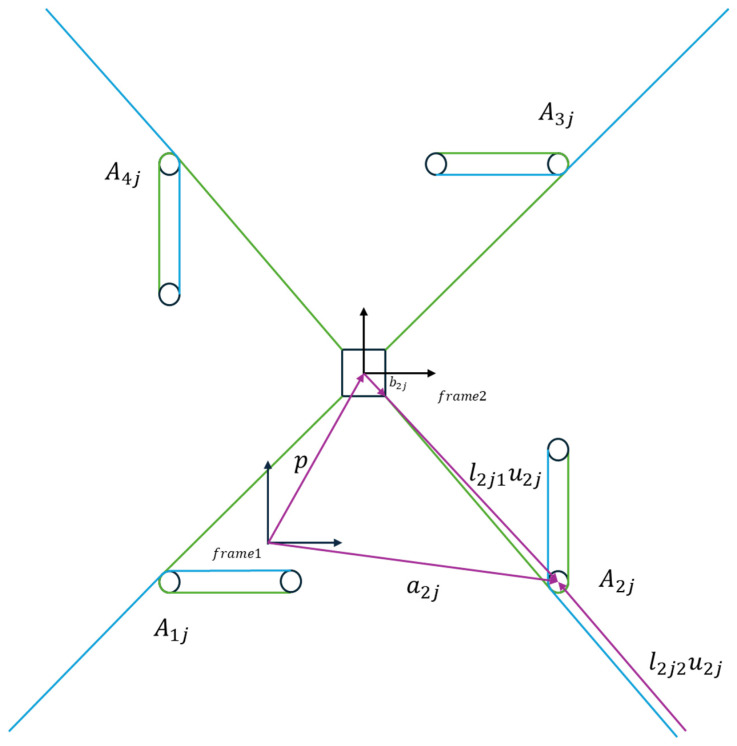
Diagram of the proposed novel planar CDPR.

The kinematics analysis in Section 3.1 establishes the positional relationships and cable length calculations, forming the foundation for precise motion control. To further understand how changes in the platform’s position and orientation influence cable velocities, the Jacobian matrix is introduced in Section 3.2. In robotic control and kinematics analysis, the Jacobian matrix plays a crucial role in describing how changes in joint space affect the end-effector’s position and orientation. By establishing a linear relationship between joint velocities and end-effector velocities, the Jacobian matrix is significant for motion control, trajectory planning, and force control in robotic systems. This study focuses on a planar CDPR with three DOFs, actuated by 12 cables. Figure 9 illustrates the details about the proposed novel planar CDPR frames and vectors. There is a fixed coordinate frame attached on the base which is named frame1, and the moving coordinate frame attached on the mobile platform is named frame2. For the i-th group of connections, let the position vector from the fixed coordinate frame frame1 to the pulley $A_{ij}$ be defined as $a_{ij}$. Furthermore, the vector from the fixed coordinate frame frame1 to the moving coordinate frame frame2 is denoted as p, and the vector from the moving coordinate frame frame2 to the connection point $B_{ij}$ on the moving platform is defined as $b_{ij}$. In addition, the cable length from $B_{ij}$ to $A_{ij}$ is $l_{ij1}$, and its direction can be represented by the unit vector $u_{ij}$. Moreover, the unit vector perpendicular from the pulley center to the cable is denoted as $d_{ij}$. Additionally, the orientation of the mobile platform relative to the fixed frame frame1 can be described by the rotation matrix R, which depends on the angle θ. Therefore, the pose of the mobile platform in the fixed frame frame1 can be described as $s={\left[\begin{array}{cc} p^{T} & \theta \end{array}\;\right]}^{T}$.

Using the definitions above, the vector of the cable can be expressed as follows:

(11) $$l_{ij1}u_{ij}=a_{ij}-p-Rb_{ij}+r_{p}d_{ij},$$

(12) $$l_{ij2}u_{ij}=\left(L_{rod}-l_{ij1}\right)u_{ij}.$$

Therefore, the effective length of the cable for $l_{ij1}$ and $l_{ij2}$ can be determined as follows:

(13) $$l_{ij1}={\left\|a_{ij}-p-Rb_{ij}+r_{p}d_{ij}\right\|}_{2},$$

(14) $$l_{ij2}=L_{rod}-{\left\|a_{ij}-p-Rb_{ij}+r_{p}d_{ij}\right\|}_{2}.$$

Since the pulley radius $r_{p}$ is very small compared to the total cable length, the deviation it introduces can likely be neglected; one of the reasons is that it has a minimal effect on the overall path. To simplify the model, we will ignore the deviation caused by the pulley in the Jacobian matrix. Therefore, the derivative of Equations (13) and (14) can be represented as follows:

(15) $${\dot{l}}_{ij1}=-\left[\begin{array}{cc} u_{ij}^{T} & u_{ij}^{T}Rb_{ij} \end{array}\right]\dot{s},$$

(16) $${\dot{l}}_{ij2}=-\left[\begin{array}{cc} u_{ij}^{T} & u_{ij}^{T}Rb_{ij} \end{array}\right]\dot{s},$$

where $\dot{s}={\left[\begin{array}{cc} {\dot{p}}^{T} & \dot{\theta } \end{array}\;\right]}^{T}$ represents the motion of the moving platform.

Since the cable $l_{ij2}$ connected to the rod does not provide any tension when it is in an extended state, it only exerts a pulling force on the rod when it is tightened. Therefore, the Jacobian matrix of this robot has two cases. When the cable $l_{ij2}$ is contracting, the Jacobian matrix of the proposed planar CDPR is represented as follows:

(17) $$J=\left[\begin{array}{cc} \begin{array}{c} u_{1a}^{T}\\ u_{1b}^{T}\\ u_{1c}^{T} \end{array} & \begin{array}{c} u_{1a}^{T}Rb_{1a}\\ u_{1b}^{T}Rb_{1b}\\ u_{1c}^{T}Rb_{1c} \end{array}\\ \vdots & \vdots \\ \begin{array}{c} u_{4a}^{T}\\ u_{4b}^{T}\\ u_{4c}^{T} \end{array} & \begin{array}{c} u_{4a}^{T}Rb_{4a}\\ u_{4b}^{T}Rb_{4b}\\ u_{4c}^{T}Rb_{4c} \end{array} \end{array}\right].$$

When the cable $l_{ij2}$ is in an extended state, the Jacobian matrix of this planar CDPR is represented as follows:

(18) $$J=\left[\begin{array}{cc} \begin{array}{c} u_{1a}^{T}\\ 0\\ u_{1c}^{T} \end{array} & \begin{array}{c} u_{1a}^{T}Rb_{1a}\\ 0\\ u_{1c}^{T}Rb_{1c} \end{array}\\ \vdots & \vdots \\ \begin{array}{c} u_{4a}^{T}\\ 0\\ u_{4c}^{T} \end{array} & \begin{array}{c} u_{4a}^{T}Rb_{4a}\\ 0\\ u_{4c}^{T}Rb_{4c} \end{array} \end{array}\right].$$

Therefore, the relationship between the mobile platform velocity and cable velocity can be represented as follows:

(19) $${\dot{l}}_{ij}=-J\dot{s}.$$

## 4. Workspace Analysis

In the analysis and design of CDPRs, the concept of workspace plays a significant role in evaluating the robot’s performance and optimizing design. Workspace analysis helps to determine the feasible regions where the end-effector can operate under specific constraints, such as cable length, tension limits, and mechanical structure. Specifically, for planar CDPRs with one rotational and two translational DOFs (1R2T), different types of workspaces are defined to describe the robot’s translational and rotational capabilities.

To evaluate the performance of a planar CDPR with 1R2T, the geometric workspace is classified into several subsets based on specific conditions and constraints [29]. These geometric workspaces can be described as follows:

(20) $$W_{T}\left(R_{0}\right)=\left\{(x,y)\in R^{2}|pose=\left(\left(x,y\right),R\right),R=R_{0}\right\}.$$

The translation workspace represents the set of all reachable positions (x,y) of the platform when its orientation is fixed at $R_{0}$. For a 1R2T robot, this workspace is typically a two-dimensional area in the plane. This definition focuses on the planar movement of the robot while keeping the platform’s orientation constant.

(21) $$W_{O}\left(\left(x_{0},y_{0}\right)\right)=\left\{R\in SO(2)|pose=\left(\left(x,y\right),R\right),\left(x,y\right)=\left(x_{0},y_{0}\right)\right\}.$$

The orientation workspace is the set of all achievable platform orientations R when the platform’s position is fixed at $\left(x_{0},y_{0}\right)$. For a 1R2T robot, this workspace corresponds to the range of rotation angles at a specific location. This workspace describes the robot’s ability to rotate its platform at a given position.

(22) $$W_{IO}\left(R_{0}\right)=\left\{(x,y)\in R^{2}|pose=\left(\left(x,y\right),R\right),R\in R_{0}\right\}.$$

The inclusion orientation workspace is the set of positions (x,y) where the platform can achieve at least one orientation within the specified set $R_{0}$. This workspace describes the positions where the robot can operate while achieving one or more target orientations.

(23) $$W_{max}=\left\{\left(x,y)\in R^{2}|pose=\left(\left(x,y\right),R\right),R\in SO(2\right)\right\}.$$

The maximum workspace represents the set of all positions (x,y) that can be reached by the platform for at least one orientation R∈SO(2). For a 1R2T robot, this workspace typically represents the robot’s largest reachable area in the plane. This workspace provides a general idea of the robot’s reachability, irrespective of specific orientation constraints.

(24) $$W_{TO}\left(R_{0}\right)=\left\{(x,y)\in R^{2}|pose=\left(\left(x,y\right),R\right),\forall R\in R_{0}\right\}.$$

The total orientation workspace is the set of all positions $\left(x,y\right)$ where the platform can achieve all orientations within a given set $R_{0}$. This workspace is stricter than the inclusion orientation workspace as it requires the platform to cover all specified orientations at every position.

(25) $$W_{D}=\left\{\left(x,y)\in R^{2}|pose=\left(\left(x,y\right),R\right),\forall R\in SO(2\right)\right\}.$$

The dexterous workspace is the set of all positions $\left(x,y\right)$ where the platform can achieve all possible orientations R∈SO(2). For a 1R2T robot, this workspace is typically very limited due to mechanical and cable constraints. This workspace reflects the robot’s ability to achieve full rotational capability at every reachable position, often making it a theoretical or highly constrained space.

To compute the translation workspace, a Monte Carlo sampling-based method was employed. Random positions within a predefined boundary were generated, and each sampled position was evaluated based on the cable length constraints. Each cable length must not exceed its maximum limit and must remain positive, and the platform orientation was assumed fixed during the calculation. In this section, a total of 100,000 random samples were evaluated to ensure sufficient resolution and coverage of the workspace.

**Figure 10:**
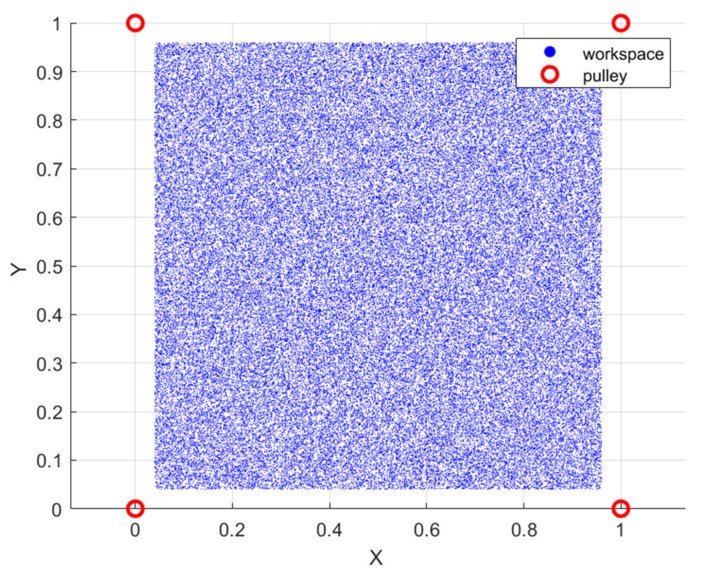
The geometric workspace of the proposed planar CDPR.

Figure 10 illustrates the translation workspace plotted in a 2D plane. Assume the proposed planar CDPR is fixed within a square frame with a side length of 1 m, each rod has a length of 1.5 m, and the cables lengths are sufficiently long. Blue dots represent the feasible positions of the platform that satisfy the cable length constraints. Red points indicate the pulleys’ positions. The workspace exhibits a square-like shape due to the symmetrical configuration of the pulleys and equal cable lengths. The dense distribution of blue dots within the workspace indicates that the platform can access nearly all positions within this region, demonstrating effective operational coverage. Under ideal conditions, the proposed planar CDPR achieves a workspace coverage of 84.64% of the theoretical maximum area. Therefore, the uniform coverage and accessibility of the workspace make this planar CDPR suitable for applications such as robotic assembly tasks, pick-and-place operations, and 3D printing. Although the workspace appears dense and accessible, practical limitations such as external disturbances may influence the actual performance. Experimental validation of the workspace will be conducted once the prototype is fully constructed to confirm the accuracy of these theoretical predictions.

**Figure 11:**
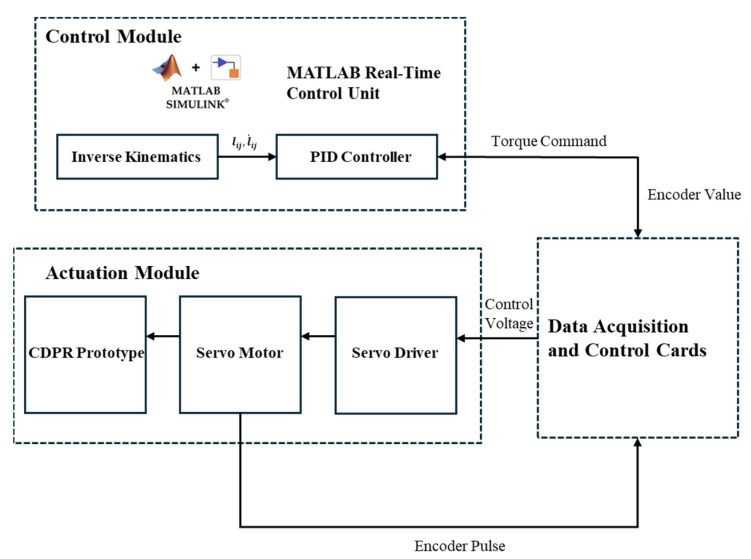
The control system diagram of the proposed planar CDPR.

The control system diagram of the proposed planar CDPR is shown in Figure 11. In the workspace of the proposed planar CDPR, the proportional–integral–derivative (PID) control method is employed to achieve high-precision positioning and trajectory tracking of the end-effector. The system architecture consists of three main components: the control module, the data acquisition and control card module, and the actuation module, forming a closed-loop real-time control system. The control module operates in the MATLAB Simulink real-time environment, where inverse kinematics is used to compute the target cable lengths, and a PID controller generates real-time torque commands to ensure precise positioning and trajectory tracking of the end-effector. Furthermore, the data acquisition and control card module connect the control module and the actuation module by transmitting control signals and collecting encoder feedback from the servo motors for real-time error correction and closed-loop control. In addition, the actuation module comprises servo drivers, servo motors, and the CDPR prototype, which adjusts cable lengths to achieve the physical motion of the end-effector.

## 5. Conclusions

In conclusion, the proposed rod–cable hybrid planar CDPR is designed and analyzed. It effectively addresses challenges in traditional cable-driven systems, such as sagging and vibration, through the integration of rigid rods for enhanced structural support. The novel planar configuration achieves stable, precise motion with three DOFs, verified through comprehensive kinematic modeling and workspace analysis. The combination of rods and cables mimics biological systems, providing an efficient and robust solution for high-precision and large-workspace applications. Future work could explore dynamic performance optimization and build a real prototype to broader applications in industrial and medical domains, for instance, implementing robust model predictive control or adaptive control algorithms to manage the nonlinearities and uncertainties inherent in the system. Additionally, we plan to incorporate additional external motors to enable the planar CDPR to move along the z-axis, thereby upgrading it to a four-DOFs system.

## Data Availability

Data are contained within the article.
